# A Pharmacoinformatics Analysis of Artemisinin Targets and *de novo* Design of Hits for Treating Ulcerative Colitis

**DOI:** 10.3389/fphar.2022.843043

**Published:** 2022-03-18

**Authors:** Rui Tian, Yufei Li, Xiaofeng Wang, Jiajun Li, Yingqian Li, Shaosheng Bei, Huashan Li

**Affiliations:** ^1^ Department of Anoenterology, Guang’anmen Hospital, China Academy of Chinese Medical Sciences, Beijing, China; ^2^ Department of Anoenterology, Xiyuan Hospital, China Academy of Chinese Medical Sciences, Beijing, China

**Keywords:** ulcerative colitis, molecular docking, molecular dynamics, MolAIcal, artemisinin

## Abstract

Ulcerative colitis (UC), as an intractably treated disease, seriously affects the quality of life of patients and has an increase in terms of incidence and prevalence annually. However, due to the lack of a direct etiology and drug-induced side effects, the medical treatment of UC falls into a bottleneck. There are many natural phytochemicals with the potential to regulate immune function in nature. Herein, a potential mechanism of artemisinin in the treatment of UC and potential druggability compounds with an artemisinin peroxide bond were discussed and predicted based on computer-aided drug design (CADD) technology by using the methods of network pharmacology, molecular docking, *de novo* drug structure design and molecular dynamics through the integration of artemisinin related targets from TCMSP, ChEMBL and HERB databases. The networks were constructed based on 50 artemisinin-disease intersection targets related to inflammation, cytokines, proliferation and apoptosis, showing the importance of GALNT2, BMP7 and TGFBR2 in the treatment of disease, which may be due to the occupation of the ricin B-type lectin domain of GALNT2 by artemisinin compounds or *de novo* designed candidates. This result could guide the direction of experiments and actual case studies in the future. This study provides a new route for the application of artemisinin and the development of drugs.

## Introduction

Ulcerative colitis (UC) is a chronic nonspecific intestinal inflammatory disease (IBD) characterized by continuous and diffuse inflammatory changes in the colonic mucosa. Its clinical manifestations are persistent or recurrent diarrhea, mucus pus and blood stool with abdominal pain and varying degrees of systemic symptoms. It was listed as an intractably treated disease by the World Health Organization. Unfortunately, in the past 20 years, the incidence and prevalence of UC have increased rapidly. Among them, North America and Northern Europe have the highest incidence and prevalence of 9–20/100,000 and 156-291/100,000, respectively (Wang and Ouyang, 2007; Ordás et al., 2012; Zhang et al., 2017).

The etiology of UC is not yet clear, but it is undeniable that the pathogenesis of UC is considered to be closely related to the imbalance of the immune response of the intestinal mucosa. The current drug treatment of UC mainly includes anti-inflammatory drugs, immunosuppressive agents and biological agents (Ordás et al., 2012). Despite its rapid onset, side effects and persistent recurrent disease usually limit the long-term use of drugs. Artemisinin is an effective ingredient extracted from the Chinese herbal medicine Artemisia annua. It is currently the most effective and low-toxicity antimalarial drug. Studies have found that artemisinin not only has antimalarial effects but is also used in the treatment of tumors, parasites, autoimmune diseases and viral infections (Yuan et al., 2017). Studies have shown that artemisinin can alleviate the levels of interleukin-6 (IL-6) and tumor necrosis factor-*α* (TNF-α) in the plasma of mice with systemic lupus erythematosus and inhibit the activities of nuclear factor‐kappa B (NF-κB) and transforming growth factor-*β*1 (TGF-*β*1) in the kidney (Wu et al., 2010). Several studies have proven that artemisinin can inhibit the activation of inflammasomes, reduce neutrophil infiltration, inhibit the function of T cells and B cells, increase the number of regulatory T cells and reduce the release of inflammatory cytokines to achieve anti-inflammatory and immunomodulatory functions (Long et al., 2016). All the evidence showed that artemisinin had the potential to treat UC, but there were no theoretical data to support it. As a compound with the potential to regulate immune function, artemisinin is expected to be used in the treatment of UC, but the relevant research is insufficient.

The application of pharmacoinformatics technology and algorithms in drug research has brought new benchmarks for identifying specific targets and screening potential new drugs (Wooller et al., 2017). It is undeniable that the innovation of new drugs was driven by pharmacoinformatics. Molecular docking, computer-assisted *de novo* drug design, pharmacokinetics and molecular dynamics simulation provide new ideas and programs for the simulation and calculation of the affinity of compounds, the development of new drugs, and the improvement of efficacy and therapeutic potential (Zhong et al., 2018). A comprehensive understanding of the molecular effects of compounds is helpful to strategically select candidate drugs and promote modern drug discovery. Modern drug databases have paid specific attention to the interaction network between chemical components and disease targets. More specifically, TCMSP and HERB, as traditional medicine databases that are constructed by the relationship between herbs, components, targets and diseases, provide strong support for the research of modern drugs and the development of phytochemicals.

This work systematically explores potential targets and pathways of artemisinin in the treatment of UC by using pharmacoinformatics technology, trying to provide a new strategy for the treatment.

## Materials and Methods

### Screening of Artemisinin and UC Related Targets

With artemisinin as the key word, the TCMSP, ChEMBL and HERB databases were used to search for potential targets of compounds. ChEMBL is a manually curated database of bioactive molecules with drug-like properties. In the target predictions module of the ChEMBL database, the species were set as human sources with a confidence interval of 90% to screen the compound-related targets. The HERB database is a high-throughput experiment- and reference-guided database of traditional Chinese medicine (Fang et al., 2021). The two modules “Related Gene Targets” and “Differentially expressed genes” in the HERB database were searched. All the obtained targets were deleted in duplicate as potential targets of artemisinin.

### Screening of Related Targets in UC

With ulcerative colitis as the keyword, the OMIM, GeneCards and DisGeNET databases were searched to obtain disease-related targets. The GeneCards database provides concise genomic-related information on all known and predicted human genes, and DisGeNET is a platform containing one of the largest publicly available collections of genes and variants associated with human diseases. The three databases are commonly used databases for disease targets (Chang et al., 2020). In the GeneCards database, setting relevance score>10, in the DisGeNET database, setting the evidence index (EI) as one means that all publications support gene-disease association (GDA) or variant-disease association (VDA) to screen disease-related targets, and finally union all targets. Cytoscape 3.7.2 was used to construct the artemisinin-UC network.

### Enrichment Analysis

To explore the mechanism of artemisinin in the treatment of UC from the perspective of biological function, the intersection target of artemisinin in the treatment of UC was used for GO and KEGG enrichment analysis based on the clusterprofiler of Rstudio, and the corrected *p* < 0.05 was set (Wu et al., 2021). The pathway was further explored and mapped from the aspects of inflammation, cytokines, proliferation and apoptosis, and the kernel targets of artemisinin in the treatment of UC were obtained.

### Molecular Docking

PubChem and Avogadro software were used to obtain the spatial three-dimensional structural formula of artemisinin, add missing hydrogen atoms, and optimize the force field of small molecules. The structural information of key targets was retrieved and predicted through the PDB database and AlphaFold Protein Structure Database (Varadi et al., 2021), respectively, and the most potential ligand binding sites were found based on cocrystals, protein cavities and literature reports. The protein and compound structures were imported into AutoDock software, setting the small molecule flexible with all bonds rotating by default and setting the central coordinates according to the ligand binding site. The Lamarckian genetic algorithm was used to evaluate the binding ability between compounds and proteins.

### De Novo Design Using the MolAIcal Tool

De novo design technology is one of the keys to constructing small molecules with the required pharmacological properties. MolAICal is programmed for 3D drug design in protein pockets with deep learning models to automatically generate effective and diverse FDA-approved drug fragments by using AutoDock Vina to evaluate the binding affinity between small molecules and proteins (Bai et al., 2021). In this study, the most stable binding protein was used as the target, the peroxide bond in artemisinin was regarded as the key pharmacophore fragment, and small molecules were constructed based on the receptor pocket structure. Two hundred automatically generated fragments were set as the father, and each time, the first 10% of the points were obtained as elitist molecules for the next iteration, with iterations 30 times or until the ligand occupied the receptor pocket. We manually evaluated the structure of *de novo* designed compounds, eliminated unreasonable small molecular structures, limited the addition of peroxy bond groups, and selected compounds with binding abilities greater than the original structure of artemisinin as candidate compounds.

### Pharmacokinetics and Drug-Likeness Analysis

Since the poor pharmacokinetics and toxicity of the candidate compounds were considered to be the main reasons for the failure of drug development, evaluating the absorption, distribution, metabolism, and toxicity of the drug was regarded as necessary. ADMETLAB2.0 (TietcheuGalani et al., 2021) was used to study the pharmacophysical properties and pharmacokinetics of candidate compounds generated by *de novo* design, including the molecular weight (MW), topological polar surface area (TPSA), QED, human intestinal absorption (HIA), human oral bioavailability>30% (F30%), plasma protein binding (PPB), blood–brain barrier (BBB), clearance (CL), human hepatotoxicity (H-HT), drug-induced liver injury (DILI), Ames test for mutagenicity (AMES), rat oral acute toxicity (ROAT), and toxic dose threshold of chemicals in humans (FNAMDD), and molecules with good absorption, distribution, metabolism and excretion were considered promising inhibitors.

### Molecular Dynamics

Gromacs was used to perform molecular dynamics simulations on candidate compounds, creating protein and compound coordinates and topology files through CHARMM36 and CGenFF, respectively (Van Der Spoel et al., 2005). The box was defined as a dodecahedron filled with water molecules, and the solution was balanced to be electrically neutral. The energy minimization and ensemble balance simulation of the system were carried out at temperature and pressure under the conditions of 300 K and 1 bar. The positions of proteins and small molecules were restricted separately, and the molecular dynamics simulation of the entire system was carried out for 10 ns.

## Result

### Related Targets of Artemisinin and UC

**FIGURE 1:**
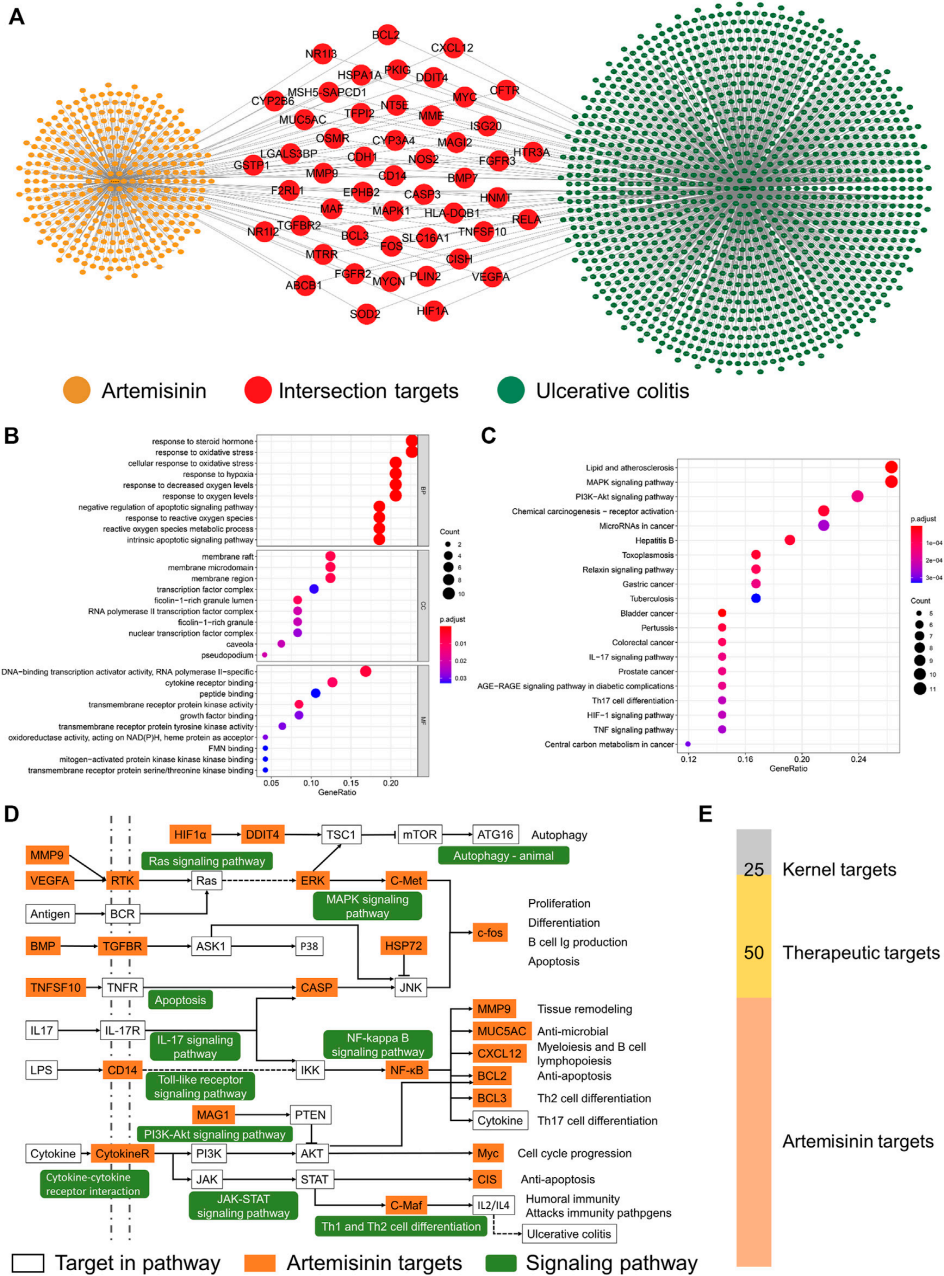
Potential biological mechanism of artemisinin in the treatment of ulcerative colitis. **(A)** Artemisinin-UC network. **(B,C)** The potential biological mechanism and pathway of artemisinin in the treatment of UC. **(D)** The therapeutic pathway of artemisinin in the treatment of UC. **(E)** The screened number of therapeutic targets.

According to the screening conditions, 15, 8, and 409 artemisinin targets were obtained from the TCMSP, CHEMBEL, and HERB databases, respectively. After deleting duplicates, a total of 413 potential therapeutic targets were obtained. The targets of UC were obtained from the GeneCards, OMIM and DisGeNET databases, and a total of 1,414 disease-related targets were obtained after union. As shown in Figure 1A, in the obtained artemisinin-related targets and ulceration-related targets, there were a total of 50 intersection targets, suggesting that artemisinin may participate in the treatment of UC through these 50 targets.

### Enrichment Analysis of the Intersection Targets

Considering the synergy between proteins, enrichment analysis of the intersecting targets will help to understand the potential biological mechanism of artemisinin in the treatment of UC. The GO enrichment results showed (Figure 1B) that the biological processes of the intersection targets were mainly enriched in the metabolism of reactive oxygen species, oxidative stress, steroid response and apoptosis. Cellular component (CC) analysis showed that the therapeutic targets were mainly concentrated in membrane rafts, membrane microdomains, membrane regions, ficolin-1-rich granules and transcription factors. The molecular functions (MFs) of the targets were enriched in the binding and activation of transmembrane receptor kinases, cytokine receptors and transcription activators. The results showed that artemisinin exerted therapeutic effects that were mainly distributed on the membrane surface and in the nucleus, participated in signal transmission between cells and regulated the synthesis and secretion of cytokines.

The results of KEGG enrichment showed (Figure 1C) that the first 30 pathways of intersecting targets mainly existed in inflammation, cell proliferation, immunity and intestinal diseases, suggesting that artemisinin exerted a therapeutic effect through multiple targets and mechanisms. To more intuitively demonstrate the pathways of artemisinin in the treatment of UC, all the enriched pathways were screened from the perspectives of inflammation, cytokines, proliferation and apoptosis, and the therapeutic pathway of artemisinin in the treatment of UC was drawn. As shown in Figure 1D, a total of 25 yellow nodes were obtained, which are the kernel targets for the treatment (Figure 1E).

### Molecular Docking Simulation of Artemisinin and Kernel Targets

**FIGURE 2:**
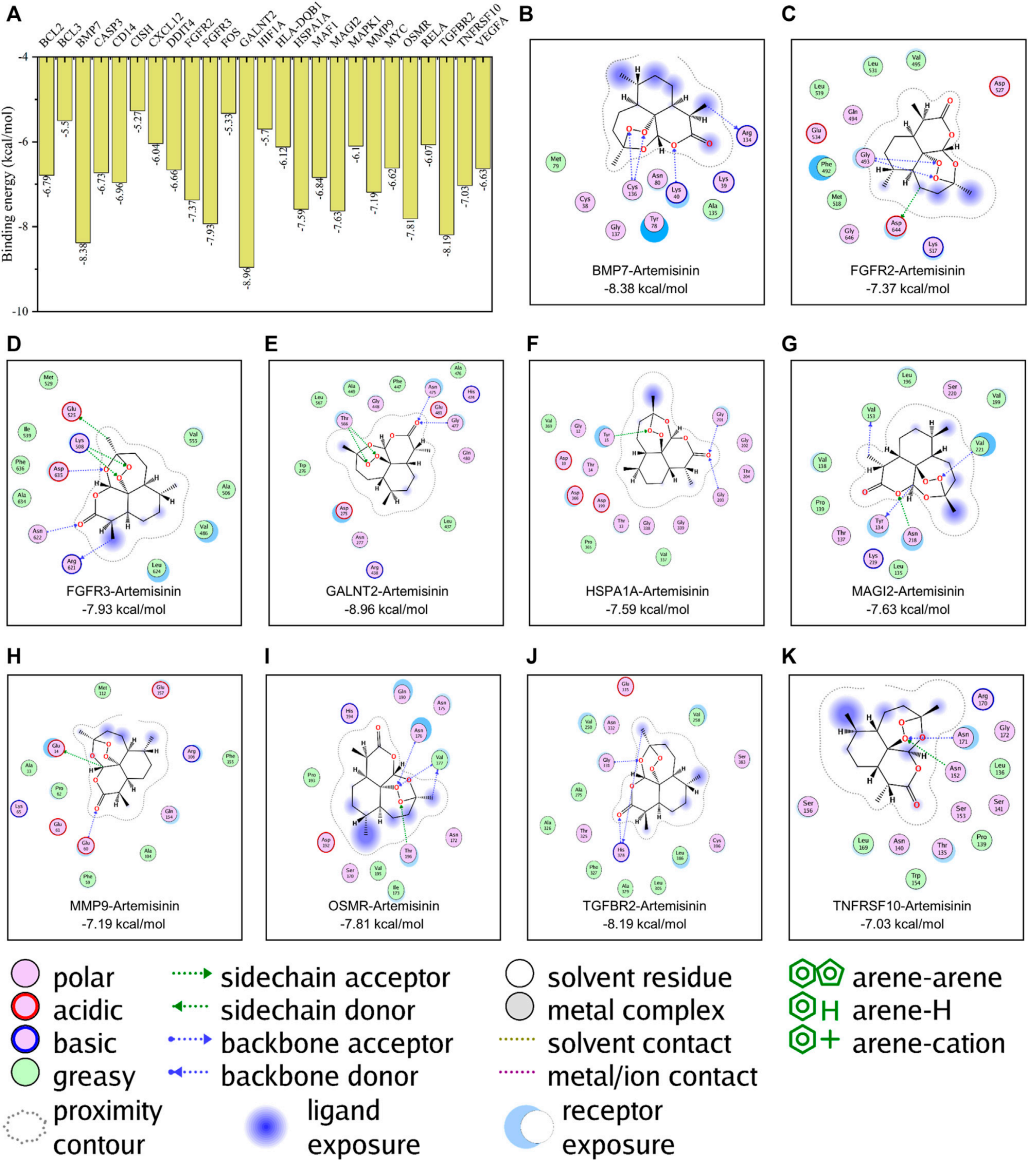
Molecular docking simulation of artemisinin with kernel targets. **(A)** The results of the binding energy between artemisinin and the protein. **(B–K)**. The docking pattern of artemisinin with the affinity top 10 kernel protein respectively.

In this study, the Lamarckian genetic algorithm was used to simulate the combination of artemisinin and the kernel targets obtained by pathway enrichment. The details of the kernel proteins are listed in Supplementary Table S1. The results showed that the binding energy between artemisinin and the protein was -5.27 ∼ -8.96 kcal/mol (Figure 2A), indicating that artemisinin can spontaneously bind to the targets. In the binding with kernel protein, artemisinin can not only form hydrophobic force but also bind to BMP7 (at residues CYS136 and ARG134), FGFR2 (at GLY483 and ASP644), FGFR3 (at LYS508, GLU525, ARG621, ASN622 and ASP635), GALNT2 (at residues ASN475, GLY477 and THR566), HSPA1A (at residues TYR15, GLY201 and GLY203), MAGI2 (at residues TYR134, VAL153, ASN218 and VAL221), MMP9 (at GLU14 and GLU60), OSMR (at ASN176, VAL177 and THR196), TGFBR2(at GLY331 and HIS328 sites) and TNFRSF10 (at ASN152 and ASN171 sites) form hydrogen bonds to increase the stability of binding (Figures 2B–K, Supplementary Figure S1 for the 3D docking pattern of molecular binding). Among them, the peroxide bond in the artemisinin structure played an important role in the process of hydrogen bond formation.

### De Novo Design of Small Molecule Compounds

**FIGURE 3:**
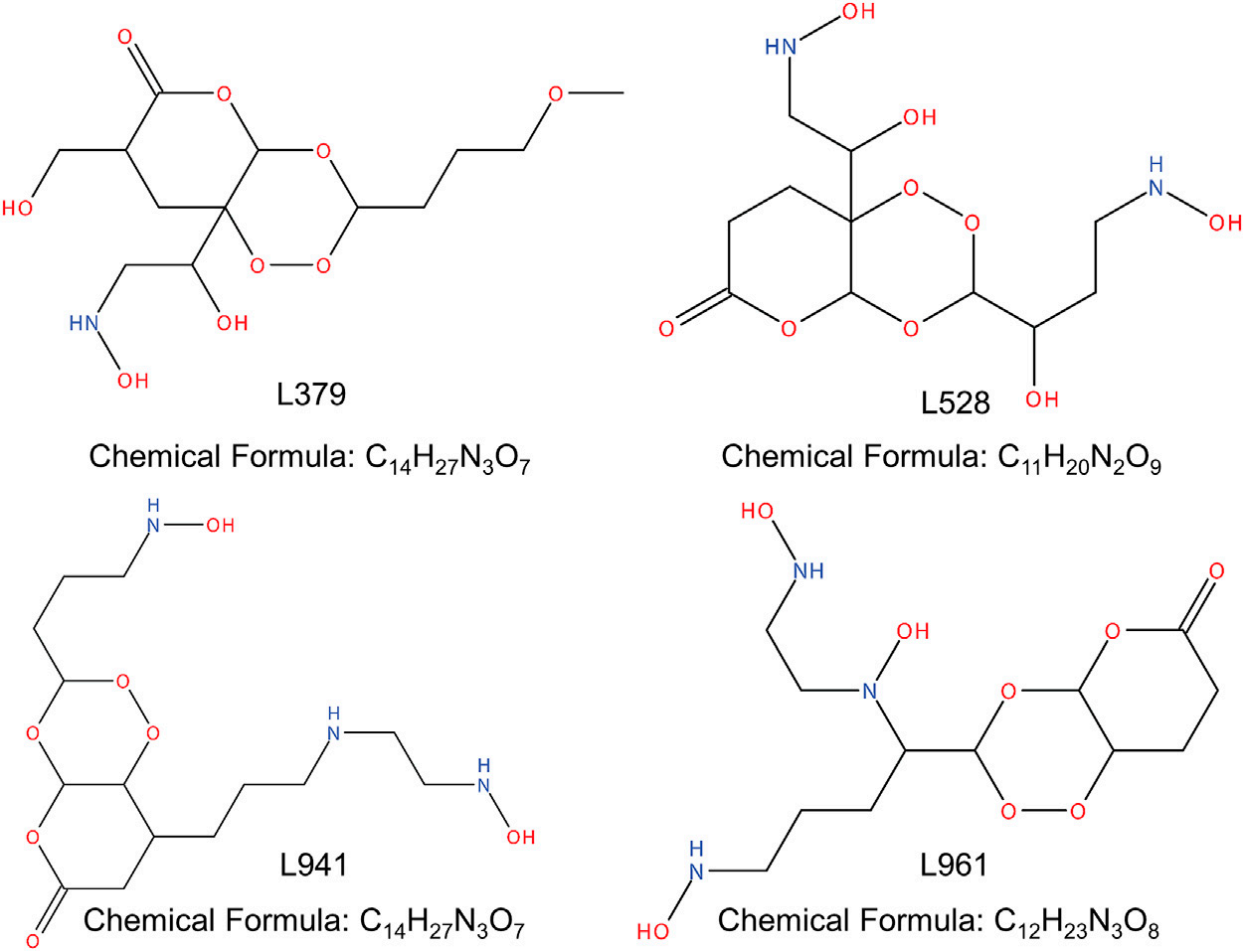
Selected *de novo* designed GALNT2 candidate compounds.

In the molecular docking simulation, artemisinin had the highest affinity with GALNT2, which was -8.96 kcal/mol. In this study, GALNT2 was selected as the receptor template, and the peroxy bond in artemisinin was used as the key pharmacophore fragment. Based on the receptor pocket structure, a *de novo* design scheme was used to construct small molecules. In the end, a total of 1,099 compounds were obtained, of which 361 were of reasonable structure (Supplementary Table S2). Using the affinity of artemisinin and GALNT2 as the threshold for further molecular screening, a total of four candidate molecules were obtained, namely, L379, L528, L941 and L961 (Figure 3). Based on topological fingerprints and the Tanimoto similarity method, RDKit was used to further calculate the similarity of candidate compounds (Bento et al., 2020). The similarities between the *de novo* designed compounds and artemisinin were more than 50%, of which L379 was 70.49%, followed by L941 (67.78%). Compared with L379, a similar chemical structure was observed from L528 with a similarity of 90.67%; however, L961 had only 53.19% similarity (Supplementary Figure S2).

### The Pharmacokinetic Parameters of *de novo* Designed Compounds

**FIGURE 4:**
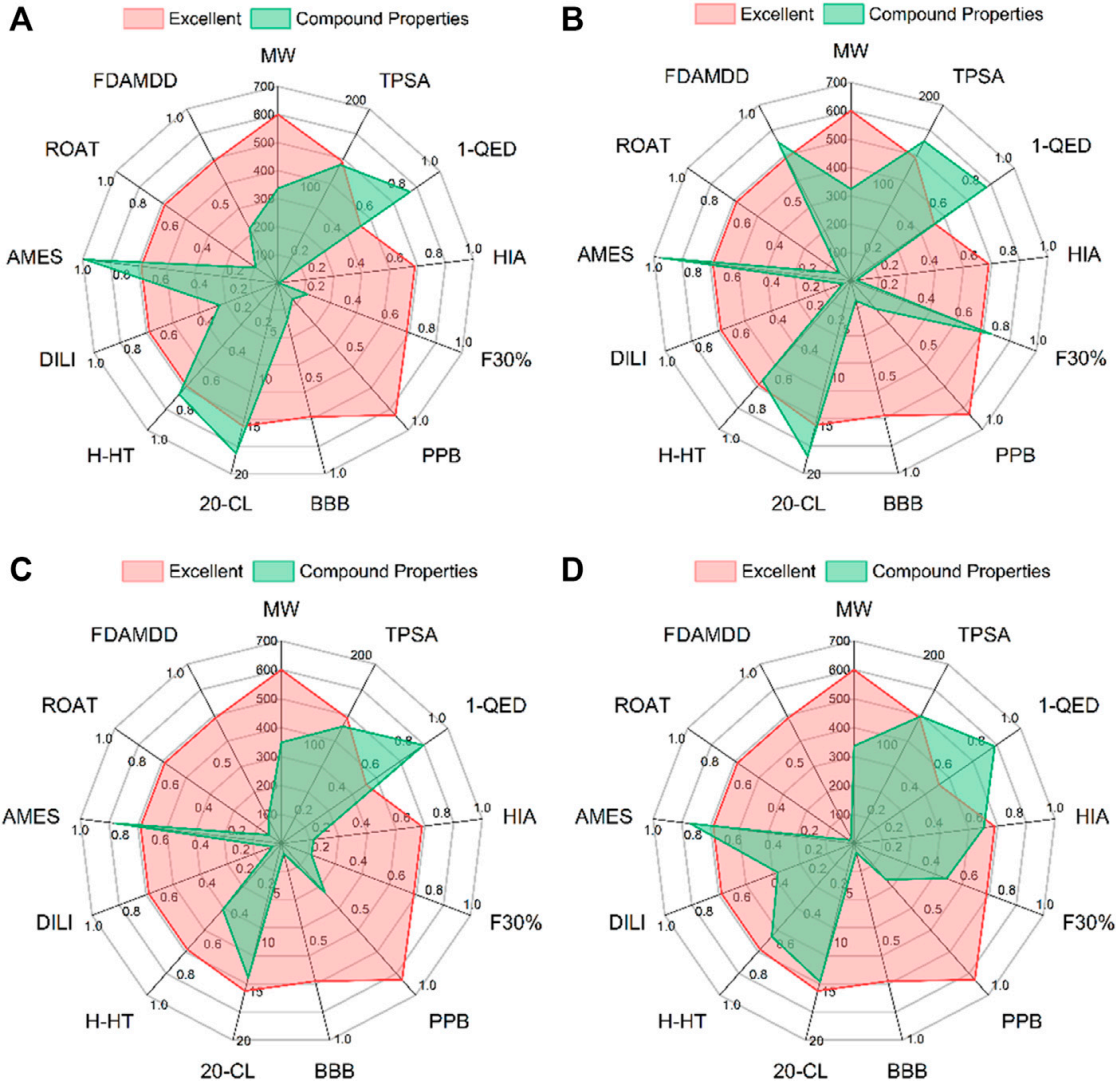
The pharmacophysical and pharmaceutical properties of the four candidate compounds. From **(A–D)** are the docking patterns of L379, L528, L941 and L961, respectively.

Having appropriate pharmacokinetic parameters and drug-like properties is the key to further drug development. ADMETLAB2.0 was used to predict the pharmacophysical and pharmaceutical properties of the four candidate compounds. As shown in Figures 4A–D, the MW of the four candidate compounds ranged from 334.12 to 349.18 g/mol. TPSA is closely related to the transport capacity of the drug in the cell. When the TPSA is greater than 140Å^2^, the penetration of the drug in the cell becomes poor (Badeliya et al., 2021). The prediction results showed that the TPSA of L379 and L941 were within the appropriate range, while the TPSA of L528 and L961 were both greater than 140 Å^2^. The absorption capacity of the drug was explained in terms of HIA and F30%. The parameters of HIA and F30% clearly indicate that each molecule had high absorption characteristics in the intestine. With the exception of L528, the oral absorption capacity of the other compounds was greater than 30%. Two parameters, PPB and BBB, were used to explore the distribution ability of candidate compounds. The results showed that the candidate compounds all have good distribution ability. The CL of the drug should be greater than 5 ml/min/kg. The results showed that the CLs of L941 and L961 were greater than this index. Toxicity is the main reason for the withdrawal of drugs from the market (Cornet et al., 2017). Five aspects of toxicity, including H-HT, DILI, AMES, ROAT and FADMDD, were evaluated, suggesting that the candidate compounds may have teratogenic effects. Among them, candidate compounds L941 and L961 have high safety, while L379 may have hepatotoxicity.

Analysis of the drug pharmacokinetics and drug-like properties showed that the candidate compounds all have the potential to become new drugs. Among them, L941 had the best result and was considered to be a promising inhibitor of GALNT2.

### Molecular Docking of Candidate Compounds

The binding energy of the candidate compounds was between −9.63 and −9.05 kcal/mol, and the affinity of L941 was the strongest. The binding conformation was further used to analyze the cause of stable binding.

**FIGURE 5:**
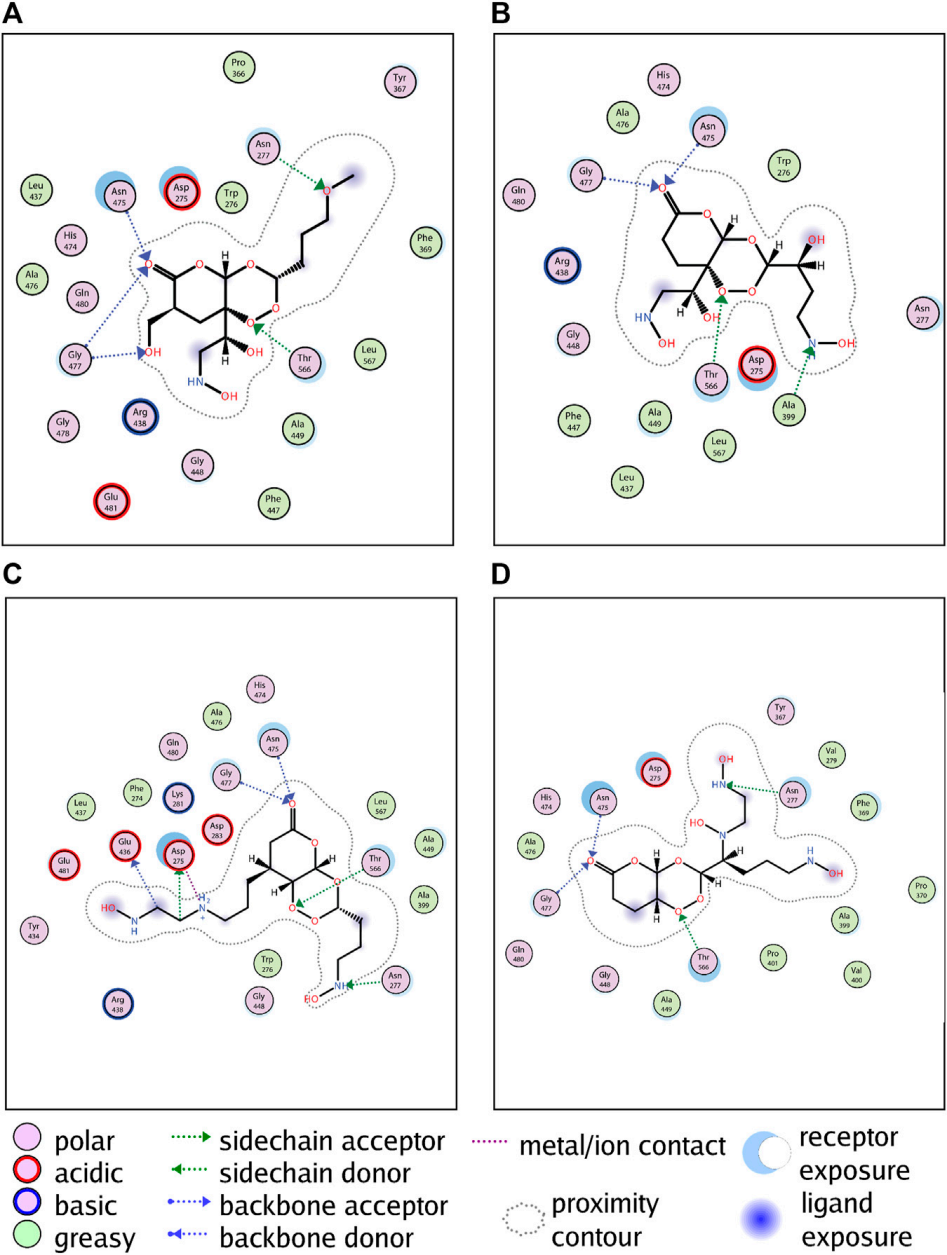
Molecular docking simulation of candidate compounds with kernel targets. From **(A–D)** are the docking patterns of L379, L528, L941 and L961, respectively.

In addition to the hydrophobic force, GALNT2 can form hydrogen bonds with L379 (Figure 5A), L528 (Figure 5B), L941 (Figure 5C) and L961 (Figure 5D) at the ASN475, GLY477 and THR566 residues, respectively (Supplementary Figure S3 for the 3D docking pattern of molecular binding). Meanwhile, GALNT2 can also form hydrogen bonds with L379, L941 and L961 at ASN277 residues to enhance the stability. L941 can also form hydrogen bonds at ASP275 and GLU436 residues, and L528 can form hydrogen bonds at ALA399 sites. In addition, L941 can also form an ionic interaction with GALNT2, making the candidate compounds more robust robustly bound to proteins.

In conclusion, compared with artemisinin, candidate molecules can form more hydrogen bonds, which is the key to stronger binding with the receptor. The molecular docking results of the four candidate compounds showed that the *de novo* designed compounds can form a tighter interaction with GALNT2, suggesting that the candidate compounds can better inhibit the activity of GALNT2.

### Molecular Dynamics of Candidate Compounds

**FIGURE 6:**
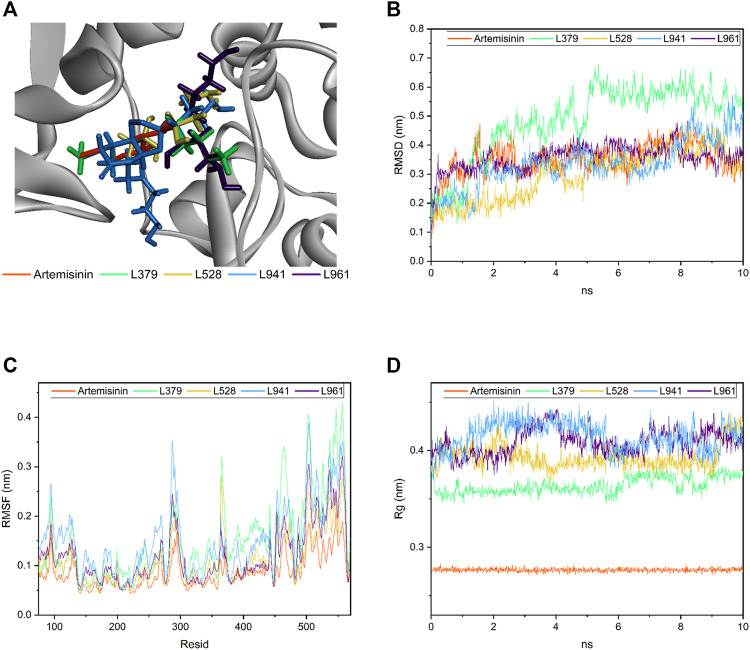
Molecular dynamics of candidate compounds. **(A)** Binding orientation and location of all four candidate compounds and artemisinin in the docking study. **(B)** The root mean square deviation (RMSD) of four candidate compounds and artemisinin. **(C)** The root mean square fluctuation (RMSF) value of residues. **(D)** The radius of gyration value in four candidate compounds and artemisinin.

In this study, artemisinin and candidate compounds were placed at the same interface. As shown in Figure 6A, both artemisinin and candidate compounds filled the receptor pocket of the protein.

Molecular dynamics is an important and effective method to evaluate molecular dynamic behavior and stability. Therefore, the binding robustness of artemisinin and candidate compounds to GALNT2 was further evaluated by this method. As shown in Figure 6B, the root mean square deviation (RMSD) value, time varying relative position between the ligand and the GALNT2 receptor pocket, of artemisinin and other candidate molecules remained in equilibrium between 10 ns, fluctuating between 0.05 and 0.48 nm, except L379. L941 and L961 had similar volatility, which may be related to their similar structures. Artemisinin and L961 fluctuated up and down at 0.35 nm after 1 ns. L941 fluctuated stably between 2 and 8 ns and in a small undulation within 8–8.5 ns. L528 fluctuated stably in 1–3 ns, increased briefly in 3.5–4 ns, and finally stabilized at 0.35 nm. Compared with other candidate compounds, L379 fluctuated greatly, reaching a peak of 0.678 nm at 5.36 ns, indicating that although the molecular docking results showed that it can produce strong affinity with proteins, its binding may not be as stable as expected over time.

The root mean square fluctuation (RMSF) value of residues was calculated to further explore the fluctuation of the backbone of the candidate compound-GALNT2 complex. As shown in Figure 6C, the results showed that each residue of GALNT2 protein had a similar fluctuation trend, peaking around residues 95, 287, 364, 465, 503 and 557, and then decreasing rapidly. Compared with the RMSF of the artemisinin-GALNT24 complex, the four candidate compounds can increase the flexibility of the receptor. In the L941-GALNT24 complex, the fluctuation of protein residues was greater, indicating that the protein structure was more flexible. The results showed that the residues involved in hydrogen bond formation generally had lower fluctuation, which may be related to the compounds occupying the receptor pocket and affecting the flexibility of residues through the formation of interaction force.

The rigidness of the compounds in the system can be addressed through inspection of the radius of gyration value. As shown in Figure 6D, the gyration radius of each candidate compound remained between 0.364 ± 0.008, 0.393 ± 0.012, 0.409 ± 0.013 and 0.415 ± 0.015 nm throughout the simulation process, and that of artemisinin fluctuated at approximately 0.28 ± 0.0018 nm. Although the fluctuation of each candidate compound was still obvious compared with the gyration radius of artemisinin, all the above values were found to be less than 0.6 nm, which undoubtedly explained why no observable deviation of the system was found throughout the simulation (Shinde et al., 2020).

Therefore, combined with the RMSD, RMSF and Rg results, except L379, other candidate compounds indisputably showed that the *de novo* designed molecules remained in the receptor pocket of GALNT2 in the dynamic state.

## Discussion

As an effective antimalarial drug extracted from Artemisia annua, artemisinin is still expected to be used in the clinical treatment of COVID-19 (NCT04387240 and NCT04801017) and schizophrenia (NCT01391403), and relevant clinical trials are ongoing. At the same time, artemisinin, as a natural compound involved in regulating immune function, is also regarded as a research hotspot in antitumor and alleviating autoimmune diseases (Gao et al., 2020; Efferth and Oesch, 2021). The incidence of UC is increasing annually and has attracted increasing attention. This study hopes to explore the possibility and potential mechanism of artemisinin in the treatment of UC from the perspective of a multitarget network of drug treatments. The results showed that there were 50 intersecting artemisinin-UC targets, of which more than half were concentrated in the inflammation, cytokine, proliferation and apoptosis pathways, suggesting that artemisinin may participate in the biological processes of reactive oxygen species metabolism, oxidative stress, apoptosis and inflammation by affecting the functions of the above targets and pathways, playing a role in treating diseases.

Studies have shown that neutrophils often infiltrate all layers of the colonic mucosa in patients with UC, resulting in the secretion of reactive oxygen species (ROS). Although the basic level of ROS can play an intestinal protective role, excessive ROS production will lead to oxidative stress and proteolytic enzyme activation, leading to endothelial cell damage, increasing the permeability of the epithelial barrier and the invasion of intestinal pathogens, and aggravating inflammatory cell infiltration and inflammatory damage, finally leading to intestinal mucosal necrosis and ulceration (Oshitani et al., 1997). Increased ROS can activate p38 MAPK and NF-κB, the molecular features of UC (Waetzig et al., 2002; Topcu-Tarladacalisir et al., 2013), mediate the expression of various proinflammatory cytokines, such as TNF-α, IL-1 and IL-8, in intestinal epithelial cells and promote the development of inflammation (Baldwin, 1996; Zhao et al., 2011).

Among the artemisinin-targeted proteins, GALNT2, BMP7 and TGFBR2 had the highest affinity, which was less than −8 kcal/mol. GALNT2 can catalyze the O-glycosylation of a variety of proteins, including the hinge region of immunoglobulin A1 and mucin, participating in the occurrence and development of inflammatory bowel diseases (Khetarpal et al., 2016; Zilmer et al., 2020). Studies have shown that GALNT2 is upregulated in ulcerative colitis patients in the active stage compared with patients in the remission period. However, GALNT2 deficiency leads to the abnormal function of mucin, which may be one of the susceptibility factors of IBD (Forgue-Lafitte et al., 2007; Nimmo et al., 2011). TNF-α is the central mediator of many autoimmune diseases, including inflammatory bowel diseases. As a kernel protein involved in the ectodomain shedding of TNF-α, GALNT2 is very important in the secretion of TNF-α (Goth et al., 2015). Artemisinin and *de novo* designed candidate compounds can stably occupy the ricin B-type lectin domain of GALNT2, inhibiting the O-glycosylation process catalyzed by the Gal/GalNAc-T motif to regulate the secretion of TNF-α.

TGFBR2 can form a heterodimer complex with TGFBR1. When stimulated by TGF-β family ligands, TGFBR2 can phosphorylate proteins and regulate cell proliferation, epithelial cell cycle arrest and wound healing. Recurrent intestinal inflammation in IBD patients induces a mucosal healing reaction, resulting in the deposition of extracellular matrix in the intestine, forming intestinal stenosis and organ failure characterized by intestinal fibrosis (Pakshir and Hinz, 2018; Lenti and Di Sabatino, 2019). Unfortunately, almost all resected UC specimens had some degree of fibrosis. Studies have shown that the activation of the TGF-β pathway is involved in the epithelial mesenchymal transformation of the intestinal mucosa, regarded as the core process of intestinal fibrosis. As an antagonist of TGFB1, BMP7, a ligand secreted by TGF-β superfamily proteins, can bind to the TGF-β receptor, recruit and activate SMAD proteins and inhibit the fibrotic changes caused by the activation of the TGF-β signaling pathway (Hao et al., 2012). In addition, it regulates monocyte chemotaxis and other specific functions (Perron et al., 2019). Artemisinin can bind to the kinase domain of TGFBR2 and affect downstream signal transmission. The differential gene expression of cell lines after treatment with artemisinin stored in the HERB database showed that the transcriptional level of BMP7 could be effectively improved (logFC = 0.574, *p* = 0.00461), suggesting that artemisinin may have certain anti-inflammatory and antifibrotic effects.

To reduce the compounds in actually screened compounds and improve the discovery efficiency of effective compounds, computer-aided drug design (CADD) was proposed, saving manpower and material resources in the research of innovative drugs. In this study, a brand-new drug was designed by using the CADD method with a *de novo* process, taking the peroxide bond, the key pharmacophore in artemisinin, as the fragment.

L941, one of the four candidate compounds screened, has stronger affinity, good pharmacokinetics and low toxicity. The results showed that in addition to the formation of hydrophobic forces, the four candidate compounds can form hydrogen bonds with GALNT2 at the ASN475, GLY477 and THR566 residues, which was identical to the bond formed by the key pharmacophore of artemisinin. In addition, the hydrogen bond acceptors and donors generated by *de novo* designed compounds can form more hydrogen bonds with GALNT2, which was the key to more robust binding.

Molecular dynamics studies showed that except for L379, other candidate compounds stably occupied the ricin B-type lectin domain of GALNT2 in the 10 ns dynamic simulation. Compared with the backbone of the artemisinin-GALNT2 complex, the complex formed by candidate compounds has better flexibility.

## Conclusion

Network pharmacology, as a combination of pharmacology and pharmacodynamics, emphasizing the integration of disease, gene, target, and drug, has been widely used for exploring the overall effect of drugs on the treatment of diseases from a macroscopic and systematic view. Deep learning is an important branch of artificial intelligence. And a *de novo* design was used to optimize the core structure of artemisinin. The results showed that artemisinin could treat UC by regulating 50 disease-related targets, reactive oxygen species metabolism, oxidative stress, apoptosis and inflammation. GALNT2, BMP7 and TGFBR2 are considered to be the kernel targets of artemisinin in the treatment of UC. Meanwhile, artemisinin and *de novo* designed candidate compounds can stably bind to the ricin B-type lectin domain of GALNT2, potentially affecting TNF-α ectodomain shedding. Although further experiments are still needed to demonstrate the aforementioned findings, this work provides a new route for the use of artemisinin in treating UC and the discovery and development of potential drugs.

## Data Availability

The original contributions presented in the study are included in the article/Supplementary Material, further inquiries can be directed to the corresponding authors.
