# Supplementary figures and images for: A Pharmacoinformatics Analysis of Artemisinin Targets and de novo Design of Hits for Treating Ulcerative Colitis
Source: Front Pharmacol. 2022 Mar 18;13:843043. doi: 10.3389/fphar.2022.843043 (PMC8971781; doi:10.3389/fphar.2022.843043)

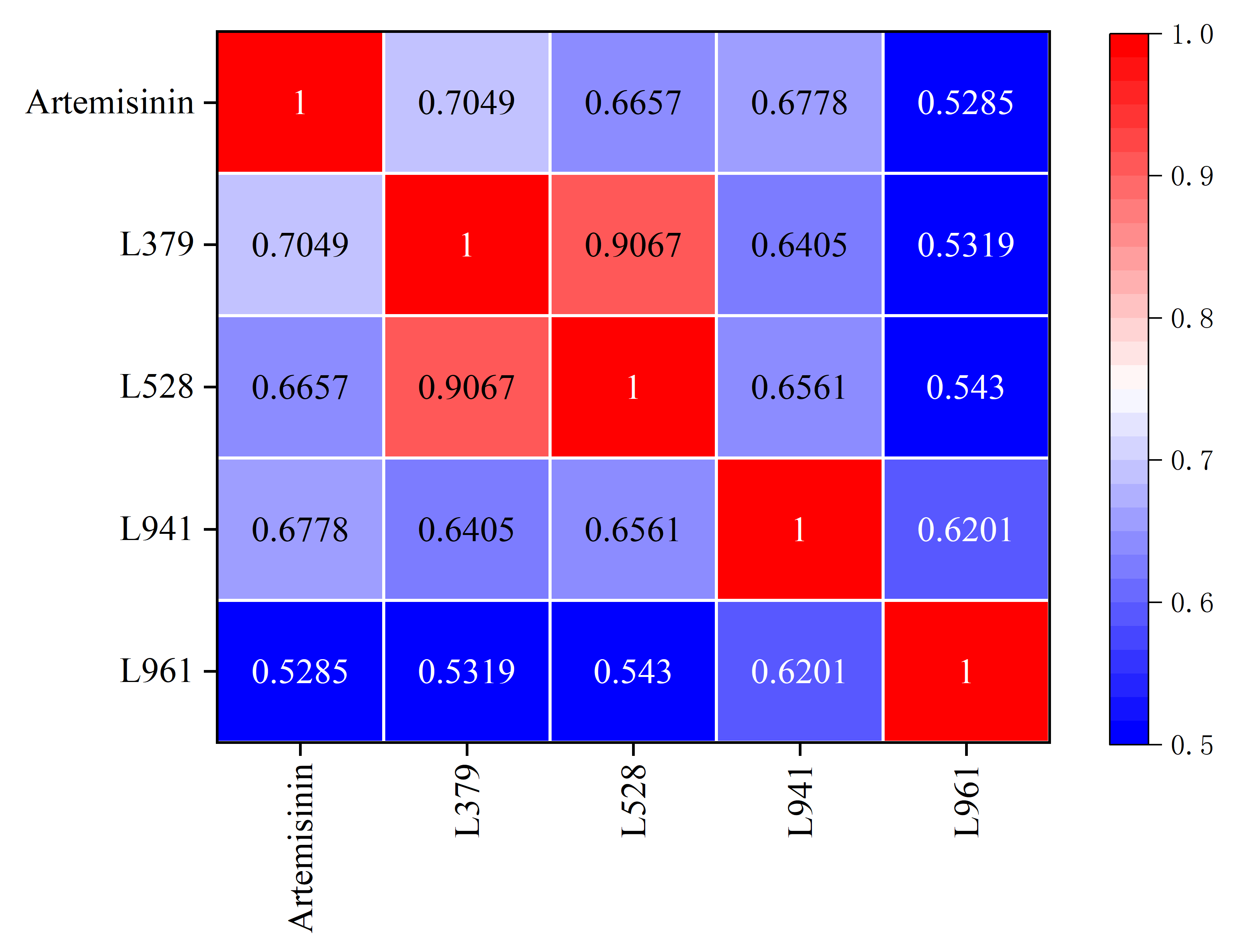

Supplement: Supplementary file 1 [file DataSheet1.ZIP › Supplementary Figure 2.tif]

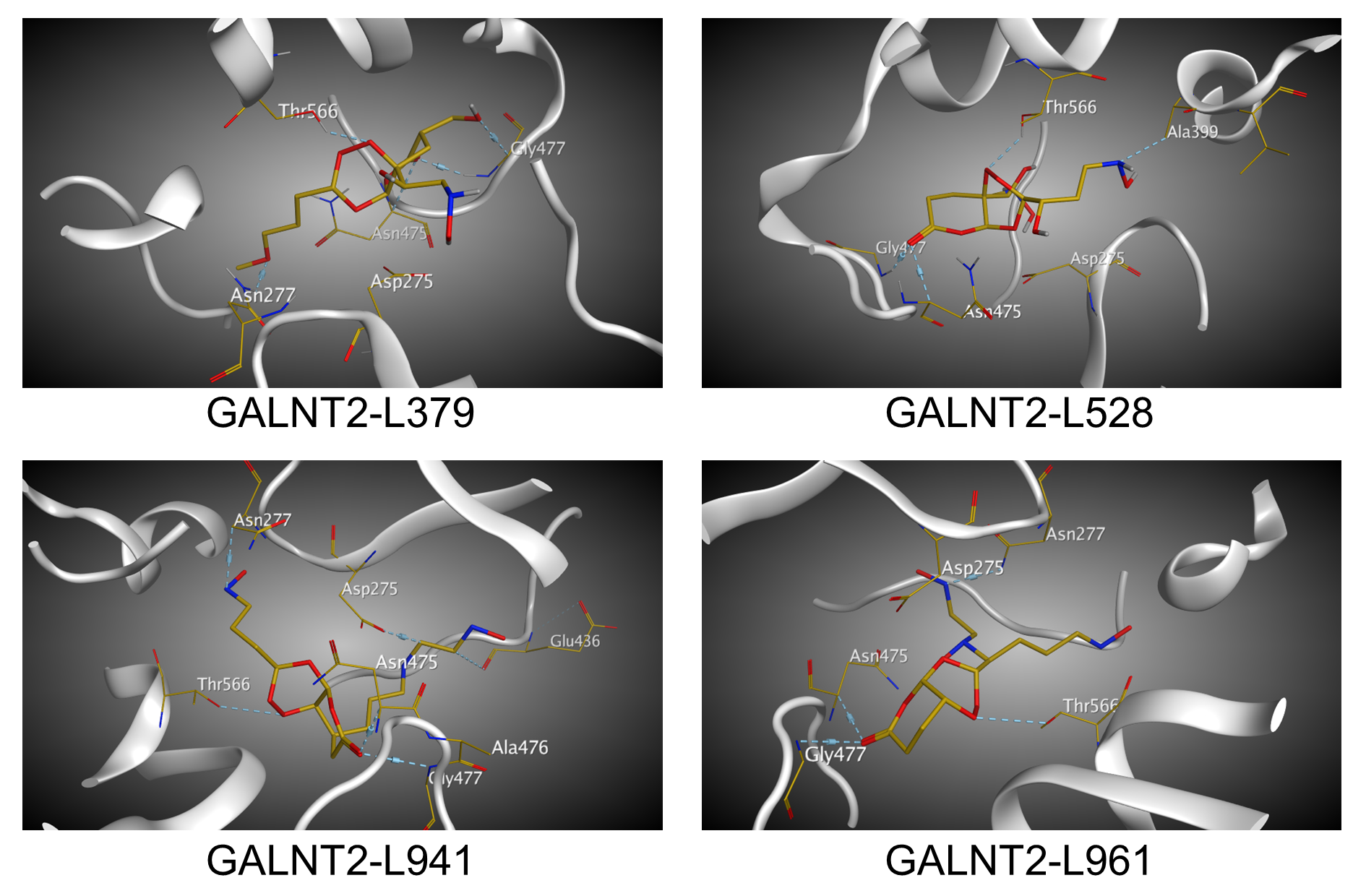

Supplement: Supplementary file 1 [file DataSheet1.ZIP › Supplementary Figure 3.tif]

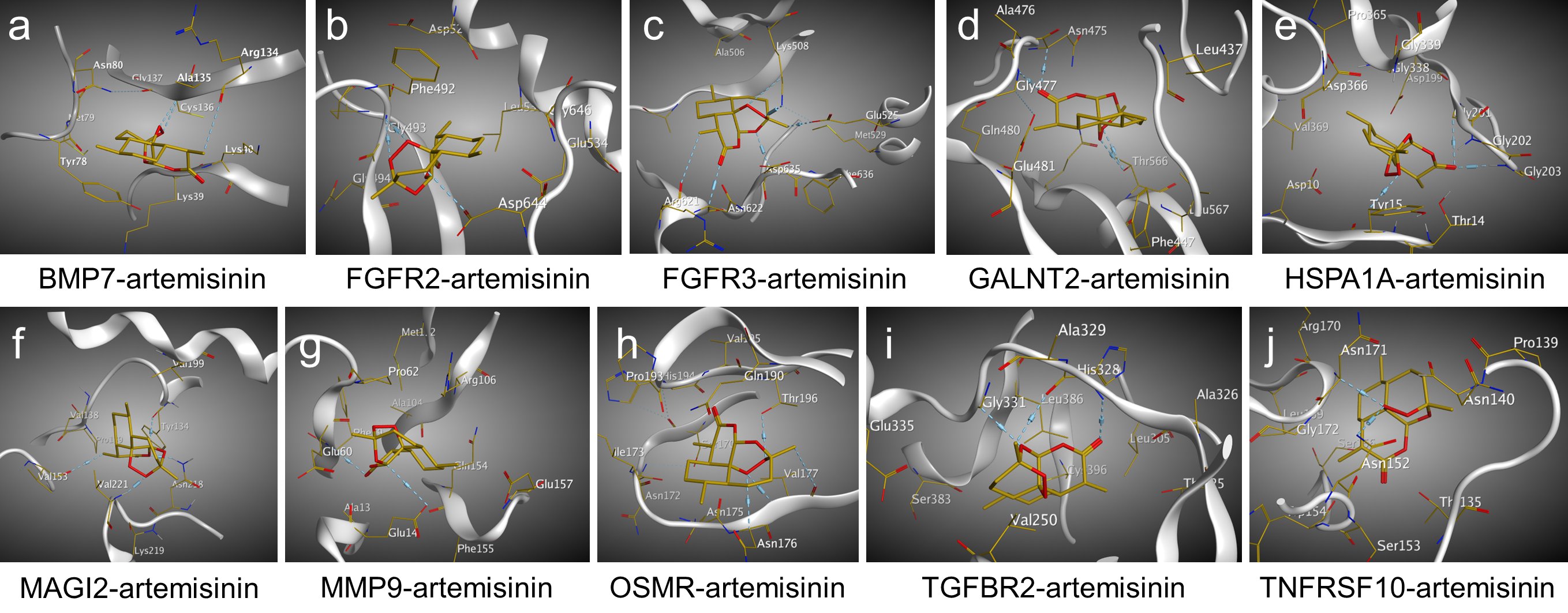

Supplement: Supplementary file 1 [file DataSheet1.ZIP › Supplementary Figure 1.tif]
